# Supplementary material for: Ketamine induces multiple individually distinct whole-brain functional connectivity signatures
Source: eLife. 2024 Apr 17;13:e84173. doi: 10.7554/eLife.84173 (PMC11023699; doi:10.7554/eLife.84173)

		PC1	PC2
1	*Cognition - Spatial Working Memory*	-0.002	0.131
2	*PANSS P1 - Delusions*	-0.157	0.330
3	*PANSS P2 - Conceptual Disorganization*	-0.267	0.024
4	*PANSS P3 - Hallucinations*	-0.022	0.335
5	*PANSS P4 - Excitement*	-0.163	0.109
6	*PANSS P5 - Grandiosity*	0.023	0.262
7	*PANSS P6 - Suspiciousness/Persecution*	-0.069	0.033
8	*PANSS P7 - Hostility*	-0.150	-0.072
9	*PANSS N1 - Blunted Affect*	-0.249	-0.062
10	*PANSS N2 - Emotional Withdrawal*	-0.295	-0.081
11	*PANSS N3 - Poor Rapport*	-0.086	-0.302
12	*PANSS N4 - Passive/Apathetic Social Withdrawal*	-0.266	-0.130
13	*PANSS N5 - Difficulty in Abstract Thinking*	-0.218	0.125
14	*PANSS N6 - Lack of Spontaneity and Flow of Conversation*	-0.277	-0.152
15	*PANSS N7 - Stereotyped Thinking*	-0.159	0.129
16	*PANSS G1 - Somatic Concern*	-0.034	0.147
17	*PANSS G2 - Anxiety*	-0.167	0.023
18	*PANSS G3 - Guilt Feelings*	-0.025	0.035
19	*PANSS G4 - Tension*	-0.197	-0.044
20	*PANSS G5 - Mannerisms and Posturing*	-0.132	0.040
21	*PANSS G6 - Depression*	-0.137	0.140
22	*PANSS G7 - Motor Retardation*	-0.236	-0.040
23	*PANSS G8 - Uncooperativeness*	-0.075	-0.369
24	*PANSS G9 - Unusual Thought Content*	-0.126	0.305
25	*PANSS G10 - Disorientation*	-0.202	0.144
26	*PANSS G11 - Poor Attention*	-0.245	-0.053
27	*PANSS G12 - Lack of Judgement and Insight*	-0.256	0.043
28	*PANSS G13 - Disturbance of Volition*	-0.197	-0.032
29	*PANSS G14 - Poor Impulse Control*	-0.059	-0.362
30	*PANSS G15 - Preoccupation*	-0.142	0.219
31	*PANSS G16 - Active Social Avoidance*	-0.250	-0.132

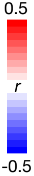

Supplement: Figure 4—figure supplement 1—source data 1. [file elife-84173-fig4-figsupp1-data1.pdf]
