# Supplementary material for: Ketamine induces multiple individually distinct whole-brain functional connectivity signatures
Source: eLife. 2024 Apr 17;13:e84173. doi: 10.7554/eLife.84173 (PMC11023699; doi:10.7554/eLife.84173)
Supplement: Supplementary file 1. — Effects were assessed using the Clinician Administered Dissociative States Scale (CADSS), the Positive and Negative Syndrome Scale for positive symptoms, negative symptoms and general psychopathology, and the Beck’s Depression Inventory (BDI). N=40. ** indicates P<.001 [file elife-84173-supp1.pdf]

| Characteristic (N=40) | Pre Ketamine | | Post Ketamine | | |
|---|---|---|---|---|---|
| | Mean | S.D. | Mean | S.D. | P-Value |
| **PANSS Positive** | 7.51 | 0.84 | 14.21 | 3.5 | <.001** |
| **PANSS Negative** | 7.95 | 1.03 | 14.57 | 5.49 | <.001** |
| **PANSS General** | 17.14 | 1.81 | 29.92 | 6.98 | <.001** |
| **PANSS Total** | 32.59 | 2.66 | 58.72 | 14.27 | <.001** |
| **CADSS** | 0.1 | 0.38 | 16.78 | 10.32 | <.001** |
| **BDI** | 1.38 | 1.93 | 0.76 | 1.53 | 0.21 |
