# Supplementary material for: Ketamine induces multiple individually distinct whole-brain functional connectivity signatures
Source: eLife. 2024 Apr 17;13:e84173. doi: 10.7554/eLife.84173 (PMC11023699; doi:10.7554/eLife.84173)
Supplement: Supplementary file 2. [file elife-84173-supp2.pdf]

Characteristic	Healthy Participants (N=40)	
	Mean	S.D.
**Age (years)**	25.73	3.74
**Gender (% male)**	0.73	-
**Participant Education (years)**	16.67	1.77
**Maternal Education (years)**	14.55	2.57
**Paternal Education (years)**	15.03	3.09
**Smoking (% smokers)**	0.03	-
**Handedness (% right)**	0.85	-
**Race (%)**	Asian: 12.5%	
	Black: 22.5%	
	White: 55%	
	Mixed: 7.2%	
	Not Specified: 2.5%	
