# Supplementary material for: Ketamine induces multiple individually distinct whole-brain functional connectivity signatures
Source: eLife. 2024 Apr 17;13:e84173. doi: 10.7554/eLife.84173 (PMC11023699; doi:10.7554/eLife.84173)
Supplement: Supplementary file 3. — For each participant we conducted a paired t-test comparing their PANSS Total score following either ketamine for placbeo administration. * significant at P<.05 uncorrected. [file elife-84173-supp3.pdf]

Participant	PANSS Total<br>Paired T-Test (Ketamine vs. Placebo)
1	*
2	*
3	*
4	*
5	*
6	*
7	*
8	*
9	*
10	*
11	*
12	*
13	*
14	*
15	*
16	*
17	*
18	*
19	*
20	*
21	*
22	*
23	ns
24	*
25	*
26	*
27	*
28	*
29	*
30	*
31	*
32	*
33	ns
34	ns
35	*
36	*
37	ns
38	*
39	*
40	ns
