# Supplementary material for: Ketamine induces multiple individually distinct whole-brain functional connectivity signatures
Source: eLife. 2024 Apr 17;13:e84173. doi: 10.7554/eLife.84173 (PMC11023699; doi:10.7554/eLife.84173)
Supplement: Supplementary file 4. — * these items have an interquartile range of 0 so any scores above or below 0 are defined as outliers. Outlier is defined as <Q1-1.5*IQR/>Q3+1.5*IQR where IQR = interquartile range, Q1=first quartile, and Q3=third quartile. [file elife-84173-supp4.pdf]

Behavioral Item	Number of Outliers<br><Q1-1.5*IQR/ >Q3+1.5*IQR
Cognition	1
PANSS P1	0
PANSS P2	1
PANSS P3	1
PANSS P4	2
PANSS P5	1
PANSS P6	0
PANSS P7	6*
PANSS N1	0
PANSS N2	0
PANSS N3	8*
PANSS N4	0
PANSS N5	1
PANSS N6	0
PANSS N7	3
PANSS G1	3
PANSS G2	0
PANSS G3	9*
PANSS G4	1
PANSS G5	0
PANSS G6	8*
PANSS G7	1
PANSS G8	4*
PANSS G9	0
PANSS G10	4*
PANSS G11	1
PANSS G12	1
PANSS G13	0
PANSS G14	3*
PANSS G15	4*
PANSS G16	1
